# Notes of Cases of Disease of the Nervous System

**Published:** 1875-10

**Authors:** 


Notes of Cases of Disease of the Nervous System in the London Hospital.—*Migraine, Chorea, and Rheumatism.*—Dr. Hughlings Jackson has been struck by the intimate relation there seems to be between chorea, migraine, and rheumatism—a relation which he believes was pointed out by the late Dr. Anstie. It is seen in several ways. Patients who have chorea are found to be subject to severe paroxysmal headache, not often, however, preceded by ocular spectra. In several recent cases of unusually severe migraine, Dr. Hughlings Jackson has found that the families of the sufferers have been subject to rheumatic fever. In patients recently admitted into the London Hospital for rheumatic fever a fair proportion have been subjected to headache, but the facts gathered from the few patients as yet interrogated are vague and inconclusive.

During a period of rather more than a year Mr. G. E. Herman worked with Dr. Hughlings Jackson on the clinical history of chorea. Notes of seventy-six cases were taken. As regards headache, it appears that fifty-three of the patients suffered from paroxysmal headaches. In four, information about headache could not be, or was not, obtained. Out of the fifty-three headaches, thirty-one were constantly attended with nausea or vomiting. In fourteen there were ocular phenomena—temporary amblyopia or spectra. In eleven there was giddiness.—*London Lancet.*

